# Aggression and courtship differences found in *Drosophila melanogaster* from two different microclimates at Evolution Canyon, Israel

**DOI:** 10.1038/s41598-019-40701-8

**Published:** 2019-03-11

**Authors:** Caroline B. Palavicino-Maggio, Séverine Trannoy, Kristina M. Holton, Xiaoying Song, Kexin Li, Eviatar Nevo

**Affiliations:** 1 Department of Neurobiology, Harvard Medical School, Boston, MA 02115 USA; 2 Research Center on Animal Cognition, Center for Integrative Biology, Toulouse University, CNRS, UPS, Toulouse, 31062 France; 3 Department of Research Computing, Harvard Medical School, Boston, MA 02115 USA; 4 Institute of Evolution, University of Haifa, Haifa, 3498838 Israel; 5 Institute of Apicultural Research, Chinese Academy of Agricultural Sciences, Beijing, 100093 China; 6 School of Life Sciences, Zhengzhou University, Zhengzhou, Henan 450001 China

## Abstract

Aggression and courtship behavior were examined of wild *Drosophila melanogaster* flies isolated from two contrasting microclimates found at Evolution Canyon in Mt. Carmel, Israel: an African-like dry tropical Slope (AS) and a European-like humid temperate Slope (ES), separated by 250 meters. Studies were carried out to ask whether behavioral differences existed between the two populations obtained from opposite slopes with divergent microclimates in Israel. First, we measured and compared *intraslope* aggression between same sex fly pairings collected from the same slope. Both male and female flies displayed similar fighting abilities from both slopes. ES males, however, from the humid biome, showed a tendency to lunge more per aggressive encounter, compared with AS males from the dry biome. Next, we tested *interslope* aggression by pairing flies from opposite slopes. ES males displayed higher numbers of lunges, and won more fights against their AS opponents. We also observed enhanced courtship performances in ES compared to AS males. The fighting and courtship superiority seen in ES males could reinforce fitness and pre-mating reproductive isolation mechanisms that underlie incipient sympatric speciation. This may support an evolutionary advantage of adaptively divergent fruit fly aggression phenotypes from different environments.

## Introduction

Aggression in *Drosophila* species has been known for over 100 years^1–3^, but only recently have fruit flies gained ground as a popular model for the study of aggression^4^. In same sex pairings of flies, both male and female *Drosophila melanogaster (D*. *mel)* exhibit aggressive behavior using gender-specific patterns of display^5^. A single gene, *fruitless*, a sex-specific transcription factor, is important in the “gender-specificity” of this behavior^6^. Fights begin with opponents approaching and orienting towards each other, accompanied by touching with the forelegs (called “fencing”). During this interaction an exchange of chemosensory information takes place while touching forelegs and then often led to a display of visual threats. Male fights then escalate to higher-intensities involving physical contact between the opponents, using patterns like “lunges” and “tussling”, after which decisions are made by the establishment of dominance in a relationship^4^. Female fights, by contrast, are less intense and most often end up with the sharing resources between flies^5^.

**Figure 1:**
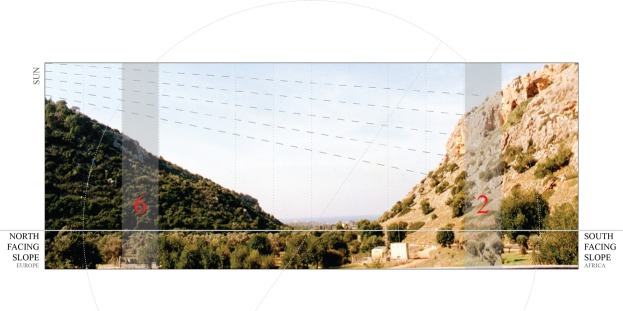
“Evolution Canyon” (EC I) microsite model in Lower Nahal Oren at Mt. Carmel, Israel. The sharp inter-slope divergence, separated by 250 meters, of savanna and forest habitats are both seen here in the cross section of EC I. The South-Facing Slope (on the right) called the African Slope (AS) receives higher levels of solar radiation yielding an African –like, hot and dry savanna climate. In contrast, the abutting, North-Facing Slope (on the left), called the European slope (ES) yields the temperate European –like, cool, humid, and forested climate. Flies for this study were obtained from collection site 2 (altitude: 90 meters above sea level) on south-facing AS slope, and collection site 6 (altitude: 90 meters above sea level) on the abutting temperate and forested north-facing ES slope. *Photograph taken by Michael Margulis from the Nevo laboratory and the picture was then modified by graphic designer*, *Jacqueline Palavicino*.

Studies focusing on the structure and evolution of aggression in natural populations in speciation, particularly incipient sympatric speciation, are rare. *D*. *mel* fruit flies are one of nine Drosophilid species found at Evolution Canyon (EC I) in Israel^7^. These populations have been suggested to be undergoing incipient sympatric speciation, or the creation of new species within a freely breeding population with gene flow^8–11^. At Evolution Canyon there are two opposing and abutting slopes within 250 meters of each other that display sharply divergent *microclimates* paralleling the *macroclimatic* divergence on a continental scale (Fig. 1). The South-Facing Slope (SFS), also known as the African Slope (AS), receives up to eight-fold higher levels of solar radiation than its counterpart. This higher solar radiation on AS causes high temperature and drought, yielding a climate and ecology resembling a dry tropical African savanna. This is in contrast to the abutting North-Facing Slope (NFS), also known as the European Slope (ES), which is temperate, forested, and has 1–7% higher humidity^12^ (Fig. 1). These sharp contrasts make EC I an ideal microsite in which to search for incipient sympatric speciation^13^.

Since the early 1990s, EC I has been explored to study adaptive evolution in action (see http://evolution.haifa.ac.il), with many studies suggesting that at this particular microsite of opposing canyon slopes, species ranging from viruses and bacteria to mammals across phylogeny appear to be undergoing incipient sympatric ecological speciation^8,13–20^. Amongst these distinct species extensively studied at EC I are *Drosophila*, showing differences in their behavior likely due to the difference in the microclimate regimes^21–23^. At EC I, populations from the AS, south-facing side of the slope are characterized by adaptive complexes against solar radiation, heat and drought savannoid stresses, while ES populations of the north-facing slope, by adaptive complexes against low solar radiation, shade, cool and humid stresses^12,13,24–27^.

*D*. *mel* has been a major model organism for studying behavior and is suggested to undergo incipient sympatric ecological speciation at EC I^7,8,10,14,15,28^. Wild *D*. *mel* populations collected from the abutting slopes - AS and ES - have revealed behavioral, phenotypic and genetic adaptations. In particular, it’s been demonstrated that AS and ES *D*. *mel* at EC I have developed adaptive fitness trait complexes of stress tolerance selected to the divergent tropical and temperate biomes that have influenced their thermal tolerance, desiccation resistance, cuticular hydrocarbons, wing shape, mate choice, reproductive activity, courtship song patterns, fecundity, fertility, and habitat choice at the opposing slopes^29–33^. A non-random mating preference for sexual partners originating from the same slope also has been observed^34^, suggesting that interslope microclimatic contrast might cause differential selection for stress tolerance-related gene complexes, along with sexual behavioral differentiation^8,10,11,14,15,30,33–39^. In addition to the phenotypic differences, whole genome sequences^37^ and its repeatome^40^ and RNA expression^41^ from *D*. *mel* also have revealed sharp interslope differences. Genomic studies have shown slope-specific differences disrupting coding sequences of genes critical for cognition, olfaction and thermoregulation, and these have been shown to be important in adaptive evolution^40,42^. Altogether, the evidence for genetic, phenotypic and behavioral divergences presented above suggest that these flies are undergoing incipient sympatric speciation potentially due to their adaptation to two distinct extreme microclimates, tropical versus temperate.

The goal of the present study was to further assess behaviors of two closely related *D*. *mel* populations of male and female fruit flies. With several long-term studies reporting genetic and behavioral differences suggesting that *D*. *mel* fruit flies are undergoing incipient sympatric ecological speciation at EC I^8,10,14,15,35^, our main objective was to ask whether these differences previously observed in *D*. *mel* collected from the two different microbiomes, might also affect complex behaviors like aggression and courtship. If so, these in turn might contribute to incipient sympatric ecological speciation.

## Results

### ES males lunge more per aggressive encounter

Aggression assays were scored by measuring individual patterns of the behavior previously observed in *D*. *mel* flies, including lunges and wing threats (males), and head butts or shorter duration wing threats (females) displayed by the flies^4,5^. First, to ask whether the microclimates at the two abutting population sites (#2 and #6, Fig. 1) in Evolution Canyon may have an effect on the levels of aggression, we measured intraslope aggression of same sex pairings of flies obtained from the same ES or AS populations (ES versus ES or AS versus AS).

**Figure 2:**
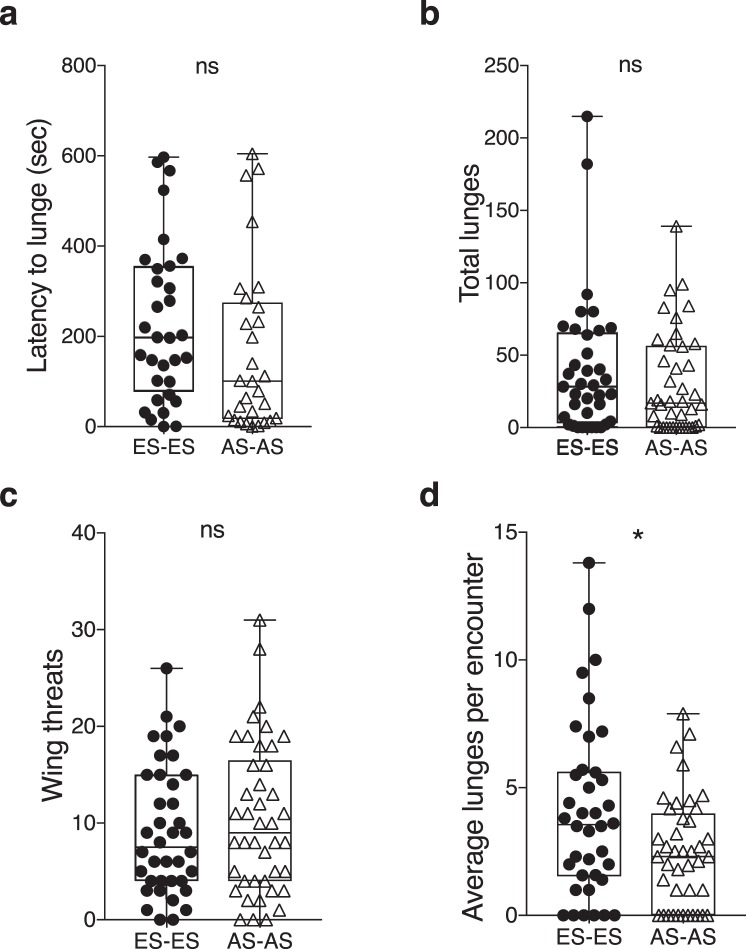
Both ES and AS male populations present the same fighting abilities. No differences were found in their (**a**) latency to lunge, (**b**) total number of lunges, or in the (**c**) total number of wing threats between ES and AS populations. However, (**d**) ES males displayed a significant increase in the average number of lunges per aggressive encounter. n = 38–42 pairs. (**a**–**d**) Center line, median; boxes, first and third quartiles; whiskers, range; circle or triangle, individual values. Individual value points on panel (**a**) represent the latency of a single fly and value points on panels (**b**–**d**) represent the counts for the number of lunges and wing threats from pairs. Statistical significance was evaluated by Mann-Whitney U-tests in all assays; *p < 0.05; ns, non-significant.

In 38 ES male fly pairings, 32 pairs engaged in aggressive interactions (84%), and in 27 ES female pairings, 24 pairs engaged in aggressive interactions (89%). In 42 AS male pairings, 30 pairs ended up in fights (71%) and in 27 AS female pairings 25 engaged in fights (93%). There were no significant differences seen when comparing ES and AS male flies in their latency to lunge (Mann-Whitney U = 579.5, *p* = 0.096; Fig. 2a) or in the total numbers of lunges (Mann-Whitney U = 862, *p* = 0.30; Fig. 2b) and wing threats observed (Mann-Whitney U = 731.5, *p* = 0.524; Fig. 2c). Small significant differences in the average numbers of lunges delivered per aggressive encounter were seen, however, and were higher in the ES population (Mann-Whitney U = 983, *p* = 0.044; Fig. 2d). In the female fight pairings, no differences were seen between the two populations in the latency to attack (Mann-Whitney U = 242.5, *p* = 0.624; Supplementary Fig. S1a), the numbers of head butts (Mann-Whitney U = 458.5, *p* = 0.105; Supplementary Fig. S1b), or the female wing threats (Mann-Whitney U = 416.5, *p* = 0.177; Supplementary Fig. S1c).

We next asked whether there were differences between these populations in their ability to establish hierarchical relationships (a “loser” is defined as the fly who retreats off the food cup away from the opponent three times in a row). In male same slope pairings, no differences were seen in the latency to establish dominance (Mann-Whitney U = 237, *p* = 0.427; Supplementary Fig. S2). Females generally do not establish hierarchical relationships during fights^5^ and that finding was confirmed in these populations as well (data not shown).

Together, the intraslope aggression experiments showed that ES and AS populations have similar fighting abilities. One difference was observed, however, in that ES males tended to lunge more per encounter than AS males (Mann-Whitney U = 983, *p* = 0.044; Fig. 2d).

### ES males are more aggressive than AS males

In the next set of experiments, we carried out interslope aggression assays using same sex pairs from opposite slopes (ES versus AS), again scoring patterns of aggression.

**Figure 3:**
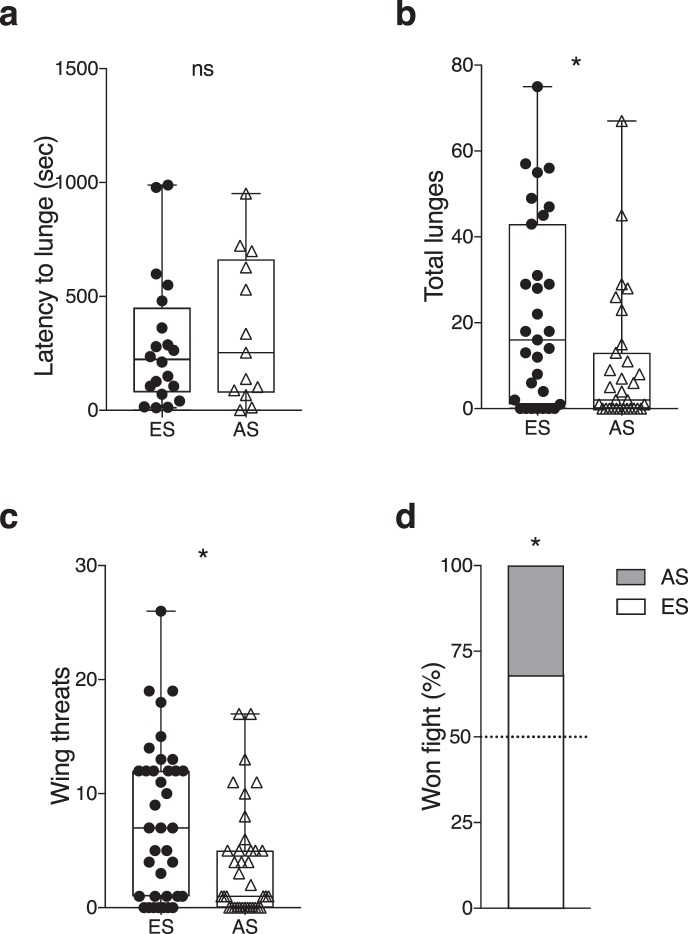
ES males have competitive advantages over AS males. (**a**) The latency to lunge was not different between ES and AS males, however, ES males displayed significantly more (**b**) number of lunges, (**c**) number of wing threats, and (**d**) won more fights against AS opponents. n = 38 individual flies. (**a**–**c**) Center line, median; boxes, first and third quartiles; whiskers, range; circle or triangle, individual values. Individual value points on panel (**a**) represent the latency of a single fly (evaluated by Mann Whitney U-test) and value points on panels (**b**,**c**) represent the counts for the number of lunges and wing threats from a single fly (evaluated by Wilcoxon matched-pairs signed-rank tests); panel (**d**) was evaluated by Chi-square test; *p < 0.05; ns, non-significant.

Interslope fights were carried out with 38 pairs of male flies from opposite slopes. In 87% of these pairs (33/38), fighting behavior was observed. ES male flies lunged first in 20 of the 33 fights (61%) (Supplementary Fig. S3a), and displayed significantly higher total numbers of lunges (Wilcoxon T = 359, *p* = 0.030; Fig. 3b) and wing threats (Wilcoxon T = 403, *p* = 0.029; Fig. 3c) against AS opponents. No differences were seen in either the latencies to lunge (Mann-Whitney U = 36.5, *p* = 0.717; Fig. 3a) or in their average number of lunges per aggressive encounter (Wilcoxon T = 245.5, *p* = 0.053; Supplementary Fig. S3b).

We also examined the establishment of hierarchical relationships and found that ES males won more fights than their AS opponents (*χ*2 = 5.785, *p* = 0.016; Fig. 3d) but no differences were detected in their latency to establish hierarchical relationships (Mann-Whitney U = 50, *p* = 0.118; Supplementary Fig. S3c). To rule out the possibility that small differences in general activity levels might account for the differences in aggression seen between the two populations, we measured locomotor activity and found no differences (Mann-Whitney U = 168.5, *p* = 0.7369; Supplementary Fig. S3d). Thus, despite the observation that little difference was seen when comparing fighting abilities between ES or AS males in same-slope fights, in interslope fights, ES males showed higher levels of aggression than AS opponents.

To examine whether these interslope effects were sex specific, we paired ES and AS females, and scored their patterns of aggression (Supplementary Fig. S4a–d). In contrast to what was observed in the male pairings, the only significant difference seen in female fights was in the numbers of female flies that attacked first. Here, ES female flies significantly attacked first when paired with AS female opponents (*χ*2 = 5.44, *p* = 0.019; Supplementary Fig. S4d).

### ES males are better courters than AS males

**Figure 4:**
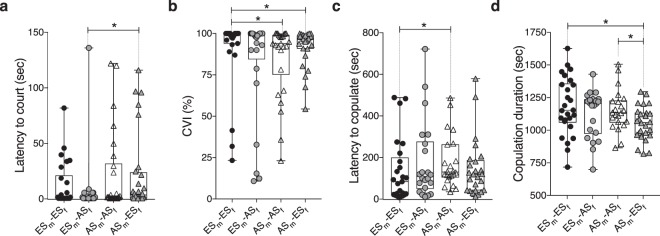
Male-female courtship behavior of pairings from ES and AS populations. (**a**) The latency to court, (**b**) Courtship Vigor Index (CVI), (**c**) latency to copulate, and (**d**) the copulation length were found different within and between ES and AS populations. n = 27–29. (**a–d**) Center line, median; boxes, first and third quartiles; whiskers, range of values; circle or triangle, individual values. Statistical significance was evaluated by Mann-Whitney U-tests for all assays; *p < 0.05; ns, non-significant.

We next assayed a second important social behavior of flies and measured courtship abilities of male flies from ES and AS slopes in pairings with females from the same (intra-) or different (inter-) slopes. These behavioral measurements included the latency to court (Fig. 4a), Courtship Vigor Index (CVI) (Fig. 4b), latency to copulate (Fig. 4c), copulation duration (Fig. 4d) and whether it was successful (Supplementary Fig. S5).

First, we asked whether any differences were seen in the time it took for males from the two slopes to initiate courtship behavior with females from the two slopes (Fig. 4a). The only differences observed in these experiments were in the different group pairings, ES males courted AS females significantly sooner than AS males courted ES females (Mann-Whitney U = 217, *p* = 0.023; Fig. 4a).

As indicators of general courtship abilities, we asked whether differences were detected in the CVI. The CVI measures the fraction of time flies engage in all aspects of courtship behavior from its initiation to copulation. Such a measure provides information about any differences in the desirability of potential mates from the two sites. We found ES males displayed a significantly higher CVI with ES females than AS males did with AS females (Mann-Whitney U = 227.5, *p* = 0.038; Fig. 4b).

We next examined the time for the initiation of copulation, its duration, and whether or not copulation was successful. We observed ES males began copulating with ES females significantly sooner than AS males did with AS females (Mann-Whitney U = 179, *p* = 0.042; Fig. 4c).

Successful copulation was achieved between 96% of the ES_m_-ES_f_ pairings (26/27), 92% ES_m_-AS_f_ pairings (23/25), 81% AS_m_-AS_f_ pairings (22/27), and 76% AS_m_-ES_f_ pairings (22/29) (Supplementary Fig. S5). When comparing the duration of copulation between a male and female flies from the same or different slopes, the duration for AS males was longer when paired with an AS female compared to when it successfully copulated with an ES female (Mann-Whitney U = 395, *p* = 0.025; Fig. 4d) and no differences were observed in copulation duration when an ES male fly copulated with either an ES or AS female. We also found copulation lasted significantly longer between ES males and ES females than it did between ES females and AS males (Mann-Whitney U = 465.5, *p* = 0.020; Fig. 4d).

These results showed that differences were found in the courtship behavior between animals from the two different slopes. ES males tended to court females more successfully and copulate with them sooner and for longer periods of time than AS males did. When females were from the ES group all parameters seemed to be slightly better, whether the males were from the ES or AS groups. This may indicate that ES females are better at mate choice preference than AS females, but further experiments would be needed to establish whether that is the case.

## Discussion

Evolution Canyon has been extensively explored since the early 1990s as an uniquely suited microsite in which to examine evolution in action, including both adaptive evolution^19,24^, and incipient sympatric speciation^13–15,43^. Multiple studies using *D*. *mel* and other *Drosophila* species found at EC I have extensively examined the biology of these fruit flies and other organisms in studies ranging from natural history and ecology, to genetics and genomics, and through to explorations of fitness and social behavior^7,8,10,11,14,15,27,30,31,33–37,40,44,45^.

The interslope gene flow of *D*. *mel* has been shown to be strongly slope-biased at EC I, 10% from the hot and dry AS to the cool and humid ES, and only 1% in the opposite direction^27^. Remarkably, however, even though *D*. *mel* can fly 16 kilometers within a single day^46^, incipient sympatric speciation may be taking place across the short distance of 250 meters that separates the sites (Fig. 1), with a tropical selection on AS, and temperate selection on ES, that seems to overrule interslope homogenization of gene flow^27^.

The objective of our study was to explore whether interslope aggression might be higher than intraslope aggression and whether this may have any evolutionary significance as a sympatric speciation factor. For these studies, we compared intraspecific aggressive patterns from within populations of fights between same sex pairings of ES or AS flies. The results showed that ES and AS male populations have essentially the same fighting abilities. They start to lunge at similar times, and display similar number of lunges and wing threats, both of which are indicators of high levels of aggression. However, we did observe an increase in the number of lunges per aggressive encounter in the ES males. These results suggest that, once engaged in fights, ES males are more aggressive. No comparable differences were found, however, in female flies.

In contrast, when we analyzed interspecific aggression between the two opposite slope populations, we found a significant effect on several indicators of aggressive behavior. ES males lunged more, displayed more wing threats and won more fights than AS males, and such differences were only seen in fights between males from the two different microclimates. Although the differences observed are relatively small, from an evolutionary stand point they may be relevant.

It is important to note that climate variables are interrelated and thus, make an association between cause and effect difficult to discern. However, low levels of humidity significantly restrict adult activity of *D*.*mel* and affect mating behavior as well as survival^47,48^. In our present study, males from the humid ES forested biome were more aggressive than males from the dry and hot AS. Our results are consistent with previous studies conducted in the 1970s and 1980s in the allopatric (or peripatric) ecological speciation of blind subterranean mole rats, *Spalax ehrenbergi*^49,50^. High territoriality and aggression seen in *Spalax* in the humid area declines gradually to low aggression in the northern Negev desert, and culminates in total pacifism and sociality seen near El Alamein in Egypt, North Africa, near the Sahara Desert^51^. The significantly higher aggression in ES male flies compared to AS males is interesting as it mirrors the local and regional *Spalax* aggression trend in which animals are more aggressive in humid and temperate climate. One can presume that more aggressive animals in the ES forested environment where there are larger populations of *Drosophila* and other insects, will benefit from their competition for better access to food, mates and territory.

Earlier studies also have shown significant differences of *D*. *mel* lines derived from the opposite slopes of the Evolution Canyon in sexual behavior^39^. These include: positive assortative mating^11,44^, interslope differences in mating propensity, sexual discrimination, reproductive behavior^29,35,45,52^, and slope specific characteristics in male’s courtship song parameters^45^. Other studies of *D*. *mel* at EC I demonstrate differences in oviposition sites where females from the AS population are more drought resistant than the ES population, and deposit eggs in accordance with their original temperature sites along a temperature gradient. These studies indicate that behavioral adaptations have evolved to cope with the sharp interslope temperature differences^31^. In the present study, we present evidence for differences in courtship abilities. We found that ES male flies tend to present better courtship performance than AS males, with a higher CVI and shorter latency to copulate. ES males also tended to successfully copulate more than AS males. However, differences were not found in any of the courtship parameters or in mating preferences when ES males were in the presence of ES or AS females. Similar results were reported in earlier studies as well^11,35,45^. The extent of courtship differentiation between the two populations in nature remains to be determined.

Previous genomic studies of these two microclimatically and ecologically contrasting populations have demonstrated that some 572 allelic loci differences were found between the populations from the opposite slopes. These were particularly involving genes concerned with responses to stimuli, and developmental and reproductive processes associated with interslope stresses^37^. Also reported are interesting results in their repeatome studies of transposable elements of *D*. *mel* at EC I^40,53^, and in more recent studies, showing gene expression and RNA editing having occurred in these populations as they adapt to the divergent interslope climates^41,54^. In particular, Nevo’s group reported that flies from the ES region had greater numbers of transposable elements than flies from the AS region, and these were associated with parallel phenotypic differences, like body size, thermoregulation, and mate choice. Nearly half of all these mobile element insertions were slope-unique, with many of them disrupting coding sequences of genes critical for cognition, olfaction and thermoregulation. All of these had been shown earlier to be important both in adaptive evolution and incipient sympatric speciation of *D*. *mel* at EC I^40^. One of the genes whose sequence was disrupted in the ES population was the gene *Cyp6a20* known to encode a Cytochrome P450 and suggested to serve a role in male-male aggression in *D*. *mel*^55^. Another gene shown to be disrupted in the same population was *roundabout1* (*robo)*, a reported axon guidance gene that is also known to serve a role in male courtship behavior^56^. While such insertions might contribute to the behavioral differences observed in this study, many further studies will be required to ask how these or additional unidentified genes might be involved in the adaptations of aggression and courtship behavior seen in flies obtained from these two microclimates.

The results of this study demonstrate that differences were observed in both aggression and courtship behavior between *D*. *mel* flies from two different microenvironments found close to each other in Evolution Canyon, Mt. Carmel, Israel. The results were consistent in that all parameters measured demonstrated that flies from the cooler, humid North-facing “European” slope were slightly more aggressive and were better courters than flies from the South-facing “African” slope. While the observations in this study support the suggestion that evolutionary processes like sympatric speciation might be taking place, further studies will be required to determine whether changes of this magnitude are also influenced by behavioral choices of flies in the wild.

## Materials and Methods

### Fly Stocks and Rearing

*D*. *mel* male and female flies were obtained from opposite mid-slopes at the EC I site on Mount Carmel, Israel in August 2016. One set of flies were collected from mid-slope #6 site of the ES slope where the microclimate is temperate, cool, humid and the slope is forested (Fig. 1). The second set of flies were collected from mid-slope #2 site on the AS slope, and its microclimate is tropical, hot, dry, and savannoid^12,24^ (Fig. 1). The flies used in our present study were representative of an average distribution of flies established from previous studies^7–12^. Isofemale lines were generated from both sites from the EC I microsite, and were reared in the laboratory in Israel for 6 generations. These isofemale lines were trapped from opposite sites and sent to Harvard Medical School, where they were reared separately for another 18 generations prior to use in experiments. Experiments were performed by two different individuals who randomly selected male and female flies from these lines for fights. Flies were reared on standard cornmeal medium in constant laboratory conditions during a 12 hours light/dark cycle at 25 °C and ambient humidity.

### Experimental set-up and design

All behavioral assays were performed in a newly developed experimental chamber that involves minimal handling of flies^57^. Males and females were reared in social isolation from late pupal, through eclosion and to the time of their use in the behavioral experiments in glass vials containing 1.5 mL of standard fly food. Behavioral assays were performed with 7-days old flies during the first 3 hours of the daily light cycle at 25 °C and 50% relative humidity.

### Aggression assays

The experimental protocol was previously described in Trannoy, *et al*.^57^. Briefly, flies were anesthetized with CO_2_ 48 hours before the behavioral assays and a dot of acrylic paint was applied on the dorsal thorax to facilitate visual tracking of individual flies. Within population (*intraslope)* or between population (*interslope*) fight assays were performed between same sex pairs of males or females from the ES or AS mid-slope populations. Food cups with standard fly food and a drop of yeast paste on the surface were used as an attractive resource in all fights. Aggressive patterns were scored during a twenty min period from the removal of the plastic divider in male-male aggression assays and during a ten min period after the first head butt in female-female assays.

### Courtship assays

Courtship assays involved male and female pairings from the same populations (ES_m_-ES_f_ or AS_m_-AS_f_) or from opposite populations (ES_m_-AS_f_ or AS_m_-ES_f_) without food resources in the experimental chamber. All singing, tapping, licking, chasing and attempts at copulation were scored for ten minutes after the first courting event.

### Quantification and Analysis

All aggression, courtship and locomotion assays were videotaped and analyzed manually.

### Aggressive behavior analysis

Lunges and wing threats were scored during a twenty min period in male-male aggression assays and head butts and shorter duration female wing threats during a ten min period in female-female aggression assays (for definition of these patterns see^5^). The latency to lunge or to head butt was defined as the time between the first encounter (social interaction lasting at least 3 seconds) on the food cup and the occurrence of the first lunge or head butt. In male-male pairings, dominance was declared after the losing fly retreated three consecutive times from the food cup following attacks from the opponent^4,5^.

### Courtship behavior analysis

All courtship behaviors including, singing, tapping, wing extension, licking, chasing and attempts at copulation were manually scored for ten min after the first courtship event. The Courtship Vigor Index (CVI) is the fraction of a ten min observation period spent by the male in courtship behavior after the onset of courtship behavior. The difference in time between when flies first interacted and started courting was defined as the latency to court and the difference between the first encounter and the onset of copulation was defined as the copulation latency.

### Locomotor behavior analysis

Locomotor activity was measured by counting the numbers of midline crosses by single flies within the first 5 min of entering behavioral chambers (22.86 mm diameter and 17 mm height).

### Statistical Analysis

All statistical analyses were performed using R statistical program (Version 1.14.4, Vienna, Austria) software and Prism7 (GraphPad Software, La Jolla, CA) for graphs. Behavioral data were analyzed with nonparametric tests. Differences in latencies to attack, lunge or establish dominance were evaluated by nonparametric two-independent-sample Mann-Whitney U-tests. For comparisons in number of lunges, average number of lunges, head butts and wing threats in *intraslope* fights, the nonparametric two-independent (unpaired)-sample Mann-Whitney U-tests were used, while for comparisons in *interslope* fights, the nonparametric Wilcoxon matched-pairs signed-rank tests was used. All courtship data were evaluated by the nonparametric two-independent (unpaired)-sample Mann-Whitney U-tests. Chi-Square tests were used to evaluate the relationship between two populations. Differences were considered statistically significant when the *p* value was less than 0.05. Outliers were tested with a Grubb’s test and the significant outliers were excluded.

## Supplementary information

Supplementary Information
