# Supplementary material for: Aggression and courtship differences found in Drosophila melanogaster from two different microclimates at Evolution Canyon, Israel
Source: Sci Rep. 2019 Mar 11;9:4084. doi: 10.1038/s41598-019-40701-8 (PMC6411990; doi:10.1038/s41598-019-40701-8)
Supplement: Supplementary file 1 — Supplementary Information [file 41598_2019_40701_MOESM1_ESM.pdf]

Aggression and courtship differences found in *Drosophila melanogaster* from two different microclimates at Evolution Canyon, Israel

Caroline B. Palavicino-Maggio[1], Séverine Trannoy[1,2], Kristina M. Holton[3], Xiaoying Song[4,6], Kexin Li[4,5] and Eviatar Nevo[4]

Supplementary Information:

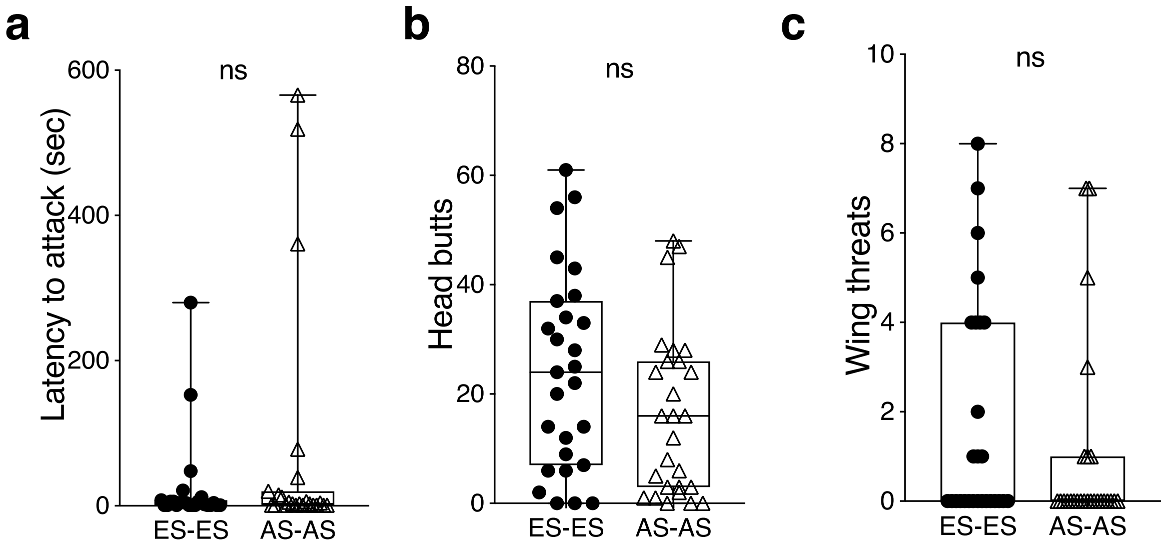

Supplementary Figure 1. Intraslope fight characterization between ES and AS females. (**a**) The latency to attack, (**b**) total number of head butts, and (**c**) the total number of wing threats were not significantly different within ES and AS female pairings. n=27 pairs. (**a-c**) Center line, median; boxes, first and third quartiles; whiskers, range of values; circle or triangle, individual values. Individual value points on panel (**a**) represent the latency of a single fly (evaluated by Mann Whitney U-test) and value points on panels (**b-c**) represent the counts for the number of lunges and wing threats from pairs (evaluated by Mann Whitney U-test);*p<0.05; ns, non-significant.

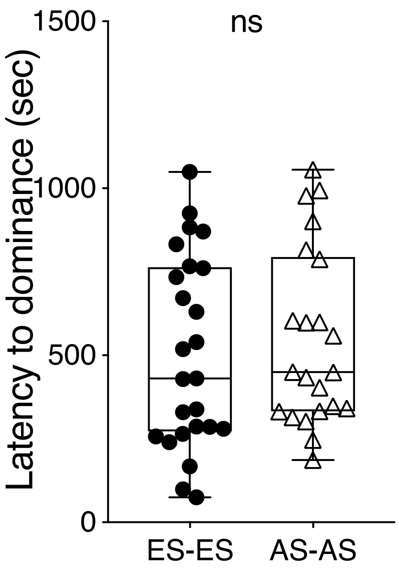

Supplementary Figure 2. The latency to dominance was not different between ES and AS pairs of male fights. n=38-42. Center line, median; boxes, first and third quartiles; whiskers, range of values; circle or triangle, individual values. Statistical significance was evaluated by Mann-Whitney U-tests; ns, non-significant.

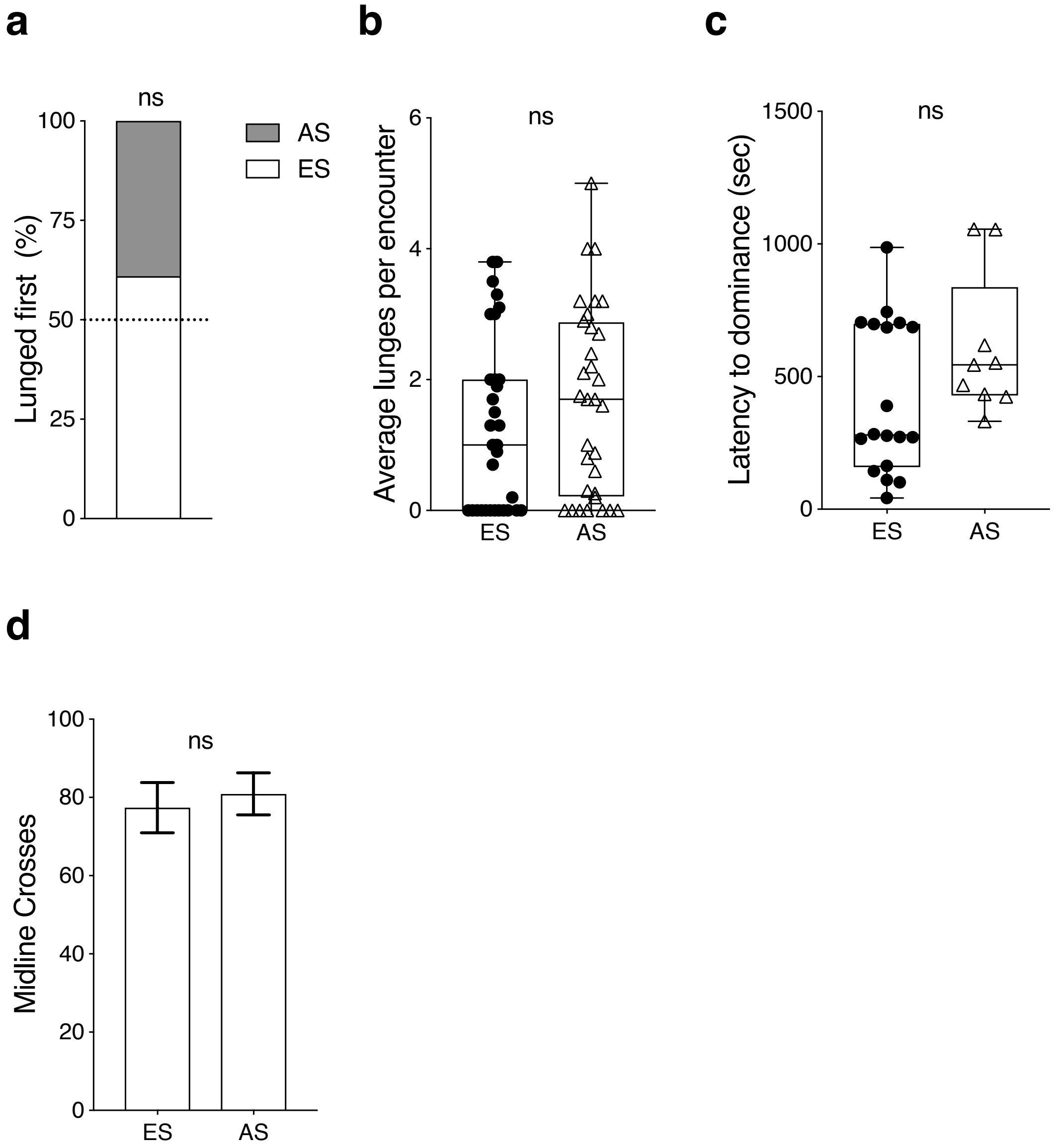

Supplementary Figure 3. Interslope male fights ES vs. AS. (**a**) The percentage of males that lunged first (evaluated by Chi-square tests), (**b**) average number of lunges per aggressive encounter, and (**c**) latency to dominance were not different (evaluated by Mann-Whitney U-tests). n=38 single flies. ns, non-significant. (**b-c**) Center line, median; boxes, first and third quartiles; whiskers, range; circle or triangle, individual values. (**d**) ES and AS males have the same levels of locomotion (evaluated by Mann-Whitney U-tests). n=19 single flies. ns, non-significant. Data are represented as means ± SEM.

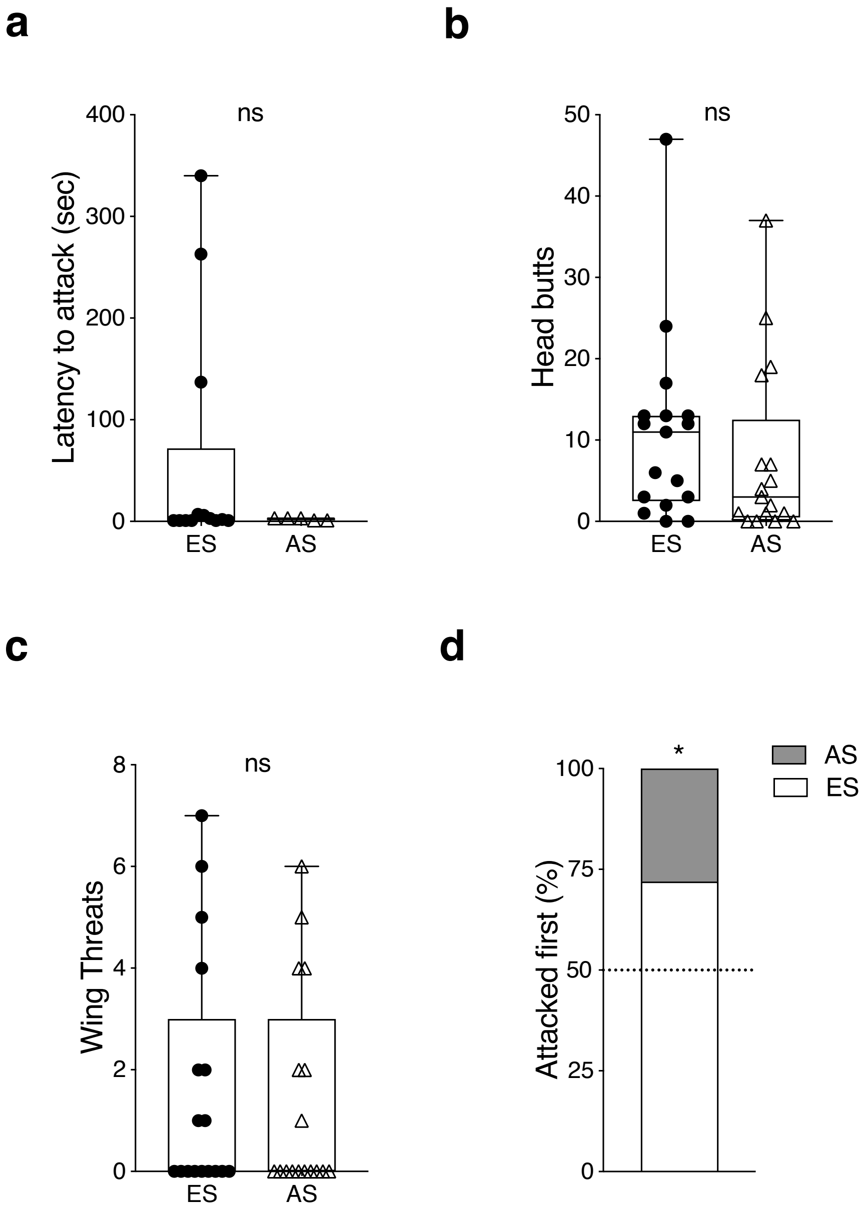

Supplementary Figure 4. Interslope female fights ES vs. AS. (**a**) The latency to attack (evaluated by Mann-Whitney U-test), (**b**) total number of head butts, and (**c**) total number of wing threats were not different (evaluated by paired sample Wilcoxon matched-pairs signed-rank tests). However, (**d**) there were many more ES females that attacked first (evaluated by Chi-square test). n=18 single flies. *p<0.05, ns, non-significant. (**a-c**) Center line, median; boxes, first and third quartiles; whiskers, range; circle or triangle, individual values.

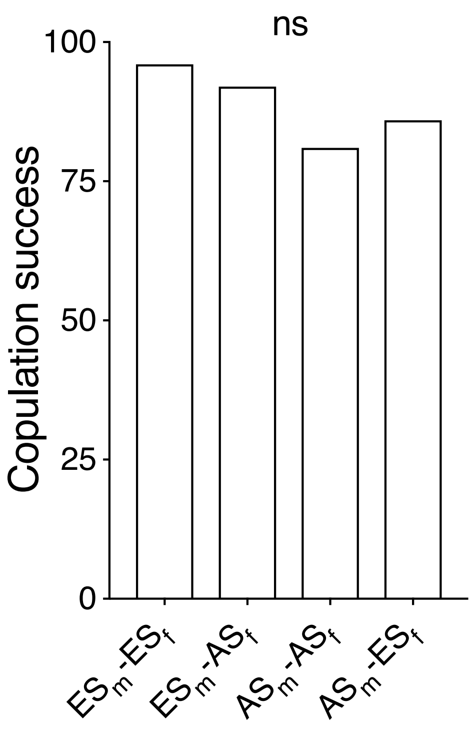

Supplementary Figure 5. The percentages of copulation success from male-female courtship pairings from ES and AS populations. n=27-29. Statistical significance evaluated by Chi-square test, ns, non-significant.
